# Supplementary material for: Triplets electrically turn on insulating lanthanide-doped nanoparticles
Source: Nature. 2025 Nov 19;647(8090):625–31. doi: 10.1038/s41586-025-09601-y (PMC12629994; doi:10.1038/s41586-025-09601-y)
Supplement: Supplementary file 1 — This file contains Supplementary Figs. 1–20, Supplementary Tables 1–4 and Supplementary Notes 1 and 2. [file 41586_2025_9601_MOESM1_ESM.pdf]

nature portfolio

https://doi.org/10.1038/s41586-025-09601-y

Supplementary information

Triplets electrically turn on insulating lanthanide-doped nanoparticles

In the format provided by the authors and unedited

Triplets electrically turn on insulating lanthanide-doped nanoparticles

Zhongzheng Yu,[1#] Yunzhou Deng,[1#] Junzhi Ye,[1,2#] Lars van Turnhout,[1] Tianjun Liu,[1] Alasdair Tew,[1] Rakesh Arul,[1] Simon Dowland,[1] Yuqi Sun,[1] Xinjuan Li,[3] Linjie Dai,[1] Yang Lu,[1] Caterina Ducati,[3] Jeremy J. Baumberg,[1] Richard H. Friend,[1] Robert L. Z. Hoye,[2] and Akshay Rao[1*]

[1] Cavendish Laboratory, University of Cambridge, Cambridge, CB3 0HE, United Kingdom
[2] Inorganic Chemistry Laboratory, University of Oxford, South Parks Road, Oxford OX1 3QR, United Kingdom
[3] Department of Materials Science and Metallurgy, University of Cambridge, Cambridge, CB3 0FS, United Kingdom
E-mail address: ar525@cam.ac.uk (A. Rao).

This file contains:
Supplementary Figures 1–20
Supplementary Tables 1–4
Supplementary Notes 1-2

Table of Contents

Supplementary Table 1 Description of typical existing SWIR LEDs without additional light-outcoupling structures. 2-4
Supplementary Table 2 Description of typical commercial SWIR laser diodes. 5
Supplementary Table 3 Ligand FTIR areas and replacement ratios. 13
Supplementary Table 4 Quantum efficiencies of LnNPs and LnNP@9-ACA nanohybrids. 17

Supplementary Fig. 1|TEM images of Nd/Yb/ErNPs. 6
Supplementary Fig. 2|HRTEM images of Nd/Yb/ErNPs. 7
Supplementary Fig. 3|XRD patterns of different LnNPs. 8
Supplementary Fig. 4| Normalized absorption of 9-ACA and NdNP@9-ACA in hexane. 9
Supplementary Fig. 5| DFT simulation of OA, 9-ACA and YbNP@9-ACA with even IR cross-section. 10
Supplementary Fig. 6|Comparison of experimental ligand on LnNPs, DFT calculated (B3LYP/6-31G+, LANL2DZ) free ligand, ligand bound to Na^{+} and ligand bound to Y^{3+}. 11
Supplementary Fig. 7 | FTIR spectra of LnNPs before and after ligand exchange. 14
Supplementary Fig. 8 | Effect of ligand exchange time. 15
Supplementary Fig. 9 | The ratio of peak emission intensity to the absorption of YbNP@9-ACA nanohybrids versus ligand exchange time. 16
Supplementary Fig. 10 | Effect of Ln^{3+} doping concentration on NIR emission. 18
Supplementary Fig. 11 | PLQEs of 9-ACA in different solvents and different LnNP@9-ACA nanohybrids in visible range. 19
Supplementary Fig.12 | TA mapping of pristine 9-ACA and LnNP@9-ACA nanohybrids. 20
Supplementary Fig.13 | PL intensity change of nanohybrids exposed to air. 21
Supplementary Fig.14 | HAADF STEM element mapping of the cross-section of YbLEDs. 22
Supplementary Fig.15 | SEM images of NdNP@9-ACA on PEDOT/ITO substrates. 23
Supplementary Fig. 16 | GIWAXS measurements of LnNPs and LnNP@9-ACA nanohybrids. 24
Supplementary Fig. 17 | NIR EL spectra versus voltage and corresponding EL mapping of LnLEDs. 25
Supplementary Fig. 18 | Visible EL spectra versus voltage for LnLEDs. 26
Supplementary Fig. 19 | Efficiency measurement of NIR LEDs. 29
Supplementary Fig.20 | Characterization of NdNP@OA, NdNP@9-ACA and pure 9-ACA LEDs. 30

Supplementary Note 1 Calculation of ligand replacement ratio. 12
Supplementary Note 2 LED Characterization. 27-28

Supplementary Table 1 Description of typical existing SWIR LEDs without additional light-outcoupling structures.

Emissive material	EL Peak (nm)	FWHM (nm)	Turn-On Voltage (V)	EQE (%)	Current Density at Peak EQE (mA cm^{-2})	Year /ref
Nd-doped GaN	1082,1364	~50	~150	-	-	2004[1]
Erbium tris(8-hydroxyquinoline)	1533	33	12	N/A	-	1999[2]
Rrbium organic compound	1540	~100	>10	N/A	<3	2000[3]
Conjugated polymer-Yb porphyrin blends	977	N/A	4	0.001	<200	2001[4]
Conjugation Platinum(II) Porphyrins	1005	>200	8.3	0.12	8-10	2011[5]
Selena-diazole containing polymers	990	>200	23.5	0.018	~20	2015[6]
D–p–A–p–D NIR chromophores	1050	220	4.8	0.33	0.1	2009[7]
D-A-D conjugated polymer	970	~150	1.1	0.05	<1	2004[8]
Single-walled carbon nanotubes	1177	50	2.1	0.01	<20	2018[9]
Y6	1040	~200	1.07	0.25	~10-100	2022[10]
Y11	1035		1.01	0.33		
IDSe-Th	1015		1.08	0.054		
Y-2W-F	1104		1.07	0.006		
SiOTIC-4F	1120		0.91	0.031		
COTIC-4F	1200		0.78	0.031		
TADF	1010	>250	3	0.003	20	2020[11]
$Yb(DBM)_3(H_2O)_2$: DPE PO	1190	-	5-6	0.15	1	2017[12]
ErQ_3 Singlet Fission OLED	1530	80	5	-	-	2018[13]
PbS in MEH-PPV	1160	190	5-6	0.27	-	2020[14]
InAs-ZnSe in MEH-PPV	1300	200	2.0	0.5	-	2002[15]
PbSe in MEH-PPV	1280	130	3.0	0.83	30	2003[16]
PbS QDs	1054	120	0.7-2.1	2.00	4	2012[17]
PbS-CdS QDs	1240	180	1.4	4.3	13.85	2015[18]
PbS in Perovskite	1391	150	<1	5.2	7.4	2016[19]
PbS QD-in-QD	1400	150	0.6	7.9	1-2	2019[20]

PbS QD-in-QD	1400	150	0.7	8	200-400	2020[21]

References

1 Kim, J. H. & Holloway, P. H. Near-infrared electroluminescence at room temperature from neodymium-doped gallium nitride thin films. Applied Physics Letters 85, 1689-1691, (2004).

2 Curry, R. J. & Gillin, W. P. 1.54 μm electroluminescence from erbium (III) tris(8-hydroxyquinoline) (ErQ)-based organic light-emitting diodes. Applied Physics Letters 75, 1380-1382, (1999).

3 Sun, R. G., Wang, Y. Z., Zheng, Q. B., Zhang, H. J. & Epstein, A. J. 1.54 μm infrared photoluminescence and electroluminescence from an erbium organic compound. Journal of Applied Physics 87, 7589-7591, (2000).

4 Harrison, B. S. et al. Near-infrared electroluminescence from conjugated polymer/lanthanide porphyrin blends. Applied Physics Letters 79, 3770-3772, (2001).

5 Graham, K. R. et al. Extended Conjugation Platinum(II) Porphyrins for use in Near-Infrared Emitting Organic Light Emitting Diodes. Chemistry of Materials 23, 5305-5312, (2011).

6 Tregnago, G., Steckler, T. T., Fenwick, O., Andersson, M. R. & Cacialli, F. Thia- and selena-diazole containing polymers for near-infrared light-emitting diodes. Journal of Materials Chemistry C 3, 2792-2797, (2015).

7 Qian, G. et al. Simple and Efficient Near-Infrared Organic Chromophores for Light-Emitting Diodes with Single Electroluminescent Emission above 1000 nm. Advanced Materials 21, 111-116, (2009).

8 Chen, M. et al. 1 micron wavelength photo- and electroluminescence from a conjugated polymer. Applied Physics Letters 84, 3570-3572, (2004).

9 Graf, A., Murawski, C., Zakharko, Y., Zaumseil, J. & Gather, M. C. Infrared Organic Light-Emitting Diodes with Carbon Nanotube Emitters. Advanced Materials 30, 1706711, (2018).

10 Xie, Y. et al. Bright short-wavelength infrared organic light-emitting devices. Nature Photonics 16, 752-761, (2022).

11 Liang, Q., Xu, J., Xue, J. & Qiao, J. Near-infrared-II thermally activated delayed fluorescence organic light-emitting diodes. Chemical Communications 56, 8988-8991, (2020).

12 Jinnai, K., Kabe, R. & Adachi, C. A near-infrared organic light-emitting diode based on an Yb(iii) complex synthesized by vacuum co-deposition. Chemical Communications 53, 5457-5460, (2017).

13 Nagata, R., Nakanotani, H., Potscavage Jr, W. J. & Adachi, C. Exploiting Singlet Fission in Organic Light-Emitting Diodes. Advanced Materials 30, 1801484, (2018).

14 Konstantatos, G., Huang, C., Levina, L., Lu, Z. & Sargent, E. H. Efficient Infrared Electroluminescent Devices Using Solution-Processed Colloidal Quantum Dots. Advanced Functional Materials 15, 1865-1869, (2005).

15 Tessler, N., Medvedev, V., Kazes, M., Kan, S. & Banin, U. Efficient Near-Infrared Polymer Nanocrystal Light-Emitting Diodes. Science 295, 1506-1508, (2002).

16 Steckel, J. S., Coe-Sullivan, S., Bulović, V. & Bawendi, M. G. 1.3 μm to 1.55 μm Tunable Electroluminescence from PbSe Quantum Dots Embedded within an Organic Device. Advanced Materials 15, 1862-1866, (2003).

17 Sun, L. et al. Bright infrared quantum-dot light-emitting diodes through inter-dot spacing control. Nature Nanotechnology 7, 369-373, (2012).
18 Supran, G. J. et al. High-Performance Shortwave-Infrared Light-Emitting Devices Using Core–Shell (PbS–CdS) Colloidal Quantum Dots. Advanced Materials 27, 1437-1442, (2015).
19 Gong, X. et al. Highly efficient quantum dot near-infrared light-emitting diodes. Nature Photonics 10, 253-257, (2016).
20 Pradhan, S. et al. High-efficiency colloidal quantum dot infrared light-emitting diodes via engineering at the supra-nanocrystalline level. Nature Nanotechnology 14, 72-79, (2019).
21 Pradhan, S., Dalmases, M., Baspinar, A.-B. & Konstantatos, G. Highly Efficient, Bright, and Stable Colloidal Quantum Dot Short-Wave Infrared Light-Emitting Diodes. Advanced Functional Materials 30, 2004445, (2020).

Supplementary Table 2 Description of typical commercial SWIR laser diodes.

Type	Emission Peak (nm)	FWHM (NM)	Operating Voltage (V)	Operating Current (mA)
L980H1	980 nm	0.5-2	2.0-2.5	300
L980P030	980 nm	10	1.5-2.0	50-70
LPS-1060-FC	1064 nm	10	1.4-2	300
LP1310-PAD2	1310	3	1-2	20-40
L1480G1	1480	20	1.6	5000-8000
LP1550-SAD2	1550	3	1-2	20-40
L1550G1	1550	20	1.5	5000-8000

All these diodes can be purchased on THORLABS, technical data can be found on the spec sheet online.

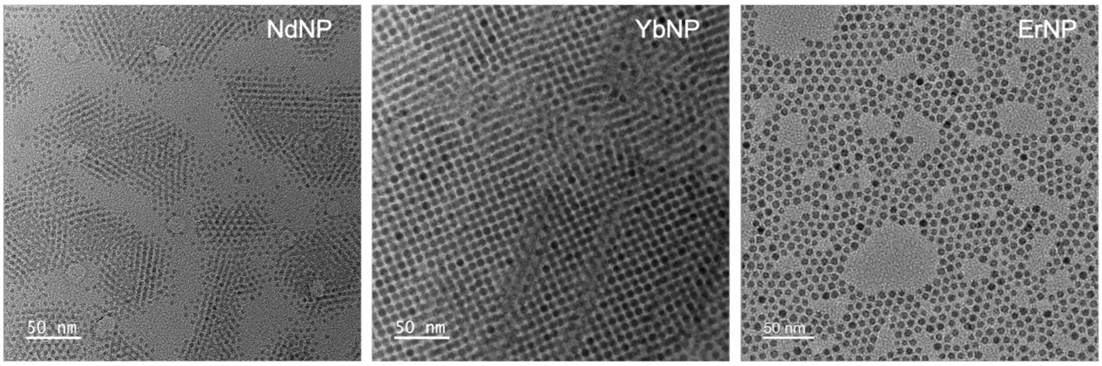

Supplementary Fig. 1 | TEM images of Nd/Yb/ErNPs.

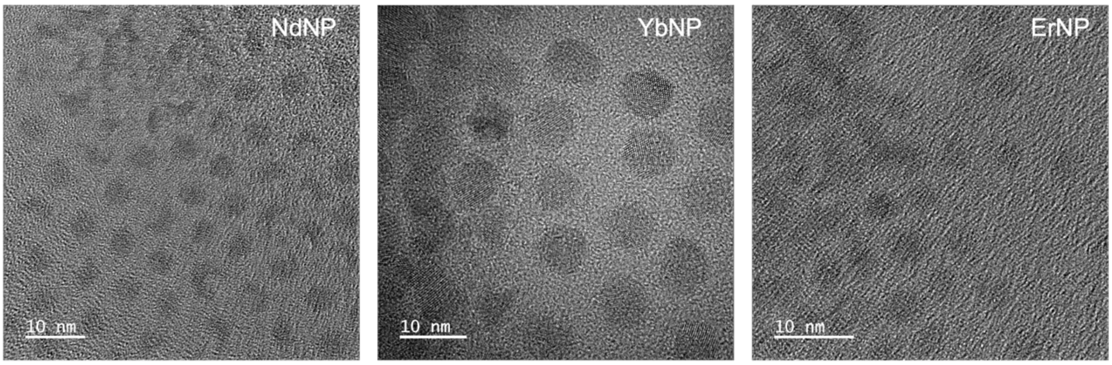

Supplementary Fig. 2 | HRTEM images of Nd/Yb/ErNPs.

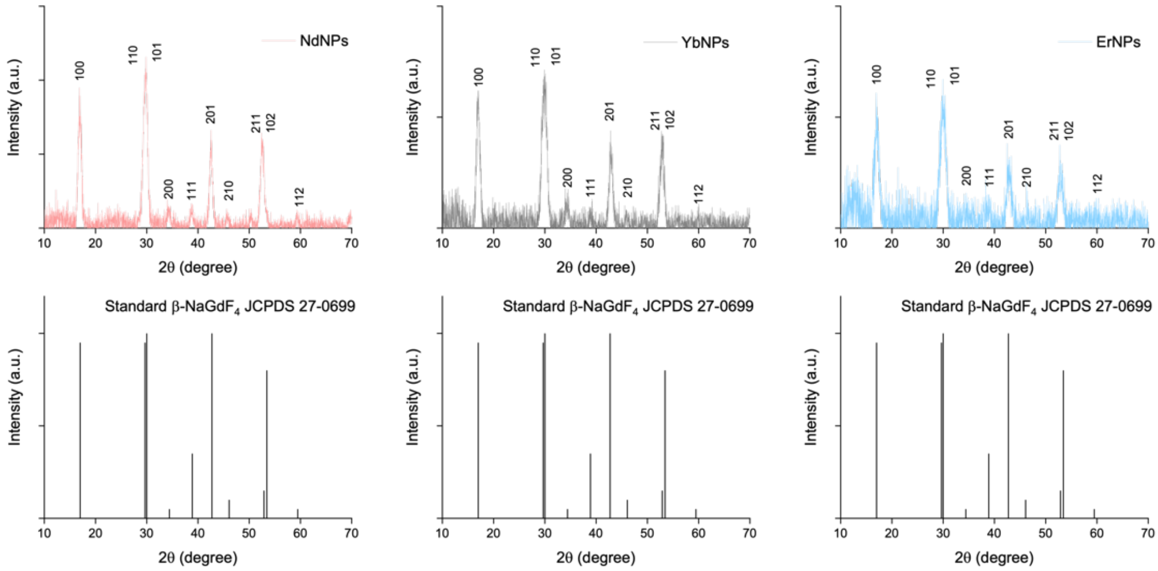

Supplementary Fig. 3 | XRD patterns of different LnNPs. The standard XRD patterns of β-$NaGdF_4$ (JCPDS 27-0699) are provided as reference.

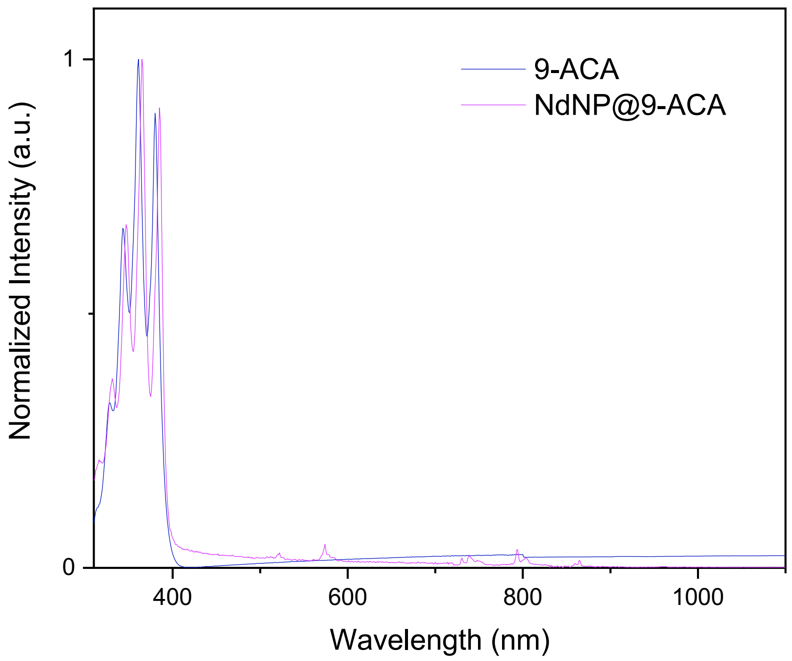

Supplementary Fig. 4 | Normalized absorption of 9-ACA and NdNP@9-ACA in hexane.

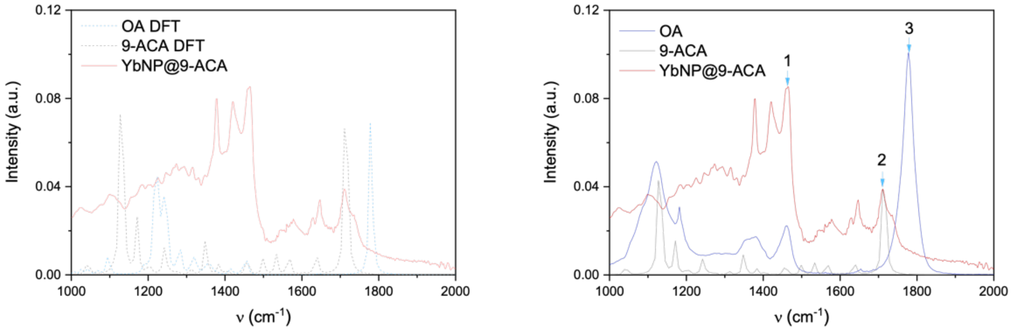

Supplementary Fig. 5 │ DFT simulation of OA, 9-ACA and YbNP@9-ACA with even IR cross-section. Peak 2 is only from 9-ACA, 1 is mix 9-ACA and OA with even IR cross-section based on DFT. So can fix OA based on intensity of 2. Peak 3 strongly suppressed as is COO^- and is removed upon binding at ~1700-1800 cm^{-1} compared to free ligands.

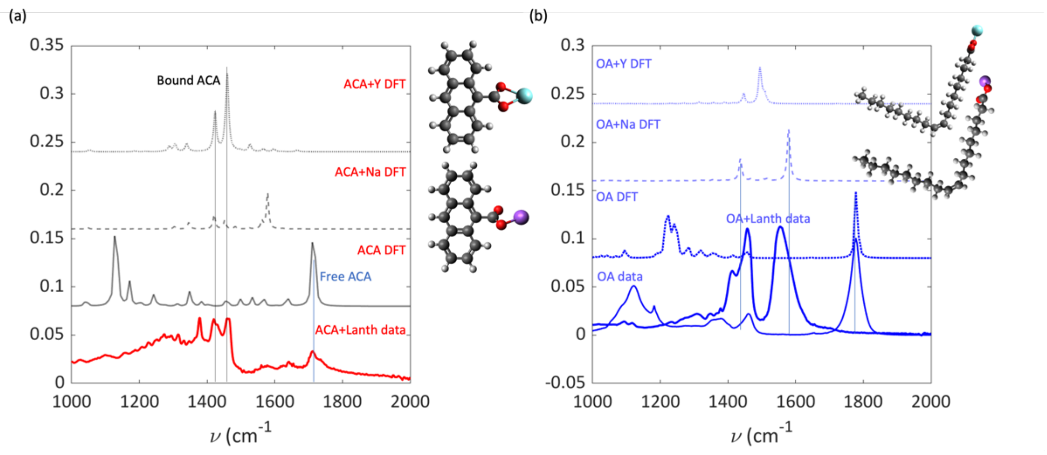

Supplementary Fig. 6│Comparison of experimental ligand on LnNPs, DFT calculated (B3LYP/6-31G+, LANL2DZ) free ligand, ligand bound to Na^+ and ligand bound to Y^{3+}. a, 9-ACA and **b,** OA. For OA bound to LnNPs, the surface ligands display vastly different IR spectra than the free ligand, which can be attributed to binding to surface Na atoms as shown by DFT calculations (Supplementary Fig. 2b). Binding to Na^+ ions enhances peaks associated with the COO atom. Contrary to previous literature [1] suggestions, the main bands at 1447 and 1495cm^{-1} are not the C=O stretch and C=C stretch but are instead both associated with the C=O stretch, with the 1495cm^{-1} peak also involving the C-H of the backbone. Replacing OA with 9-ACA results in similar changes, where the free 9-ACA ligand displays a very different IR spectrum to surface bound 9-ACA. The enhancement of peaks associated to COO^- at 1450 cm^{-1} is again observed. Combined with Fig. 2 c and d, 9-ACA molecules likely binding to Y^{3+} ions on the surface, unlike with OA, as binding to Na^+ does not result in similar changes in the DFT spectrum.

Supplementary Note 1 Calculation of ligand replacement ratio

Determining the replacement ratio is first done by normalizing the OA-conjugated and 9-ACA-replaced device lanthanide spectra to the OA C-C stretch mode at ~1595 cm^{-1}. We then subtract both spectra to yield the red curve corresponding to the signatures due to ligand replacement of OA by 9-ACA. The blue shaded area corresponds directly to the ~1595 cm^{-1} C-C stretch mode of the OA ligand. The red shaded region corresponding to the main 9-ACA C=C backbone stretch coordinated to a ligand, which can be confirmed by comparison to the fully replaced 9-ACA ligand spectrum above it in **Extended Data Fig. 1**.

We then normalize for the different oscillator strength of these ligands by comparison to the DFT predicted spectrum, to give a normalized ratio between the OA and 9-ACA oscillator strength of f_{ACA}= 1.48. The area under each shaded region (A_{ACA} in red and A_{OA} in blue) may then be divided to obtain the replacement ratio R, with exact values listed in Supplementary Table S3.

$$R = \frac{A_{ACA}/f_{ACA}}{A_{ACA}/f_{ACA} + A_{OA}}$$

Supplementary Table 3 Ligand FTIR areas and replacement ratios.

Ln^{3+}	9-ACA FTIR area (A_{ACA})	OA FTIR area (A_{OA})	Replacement ratio (R)
Er	0.70	12.6	3.6%
Nd	0.58	5.43	6.8%
Yb	0.16	10.2	1.0%

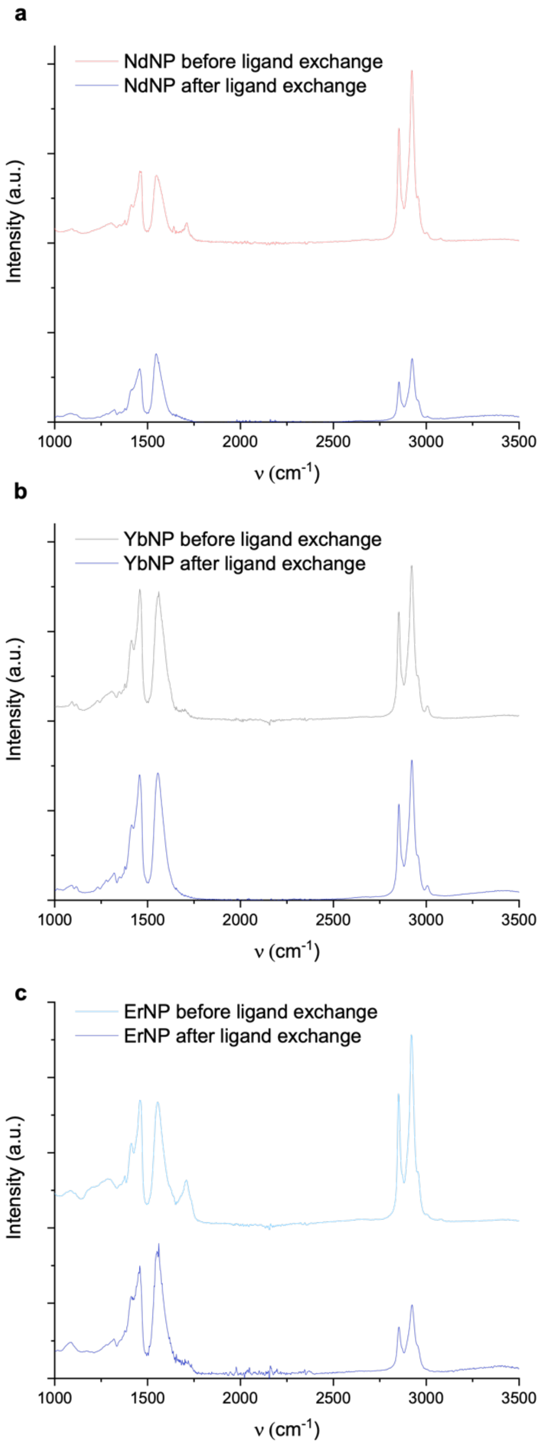

Supplementary Fig. 7 | FTIR spectra of LnNPs before and after ligand exchange. a, NdNPs; **b,** YbNPs; and **c,** ErNPs.

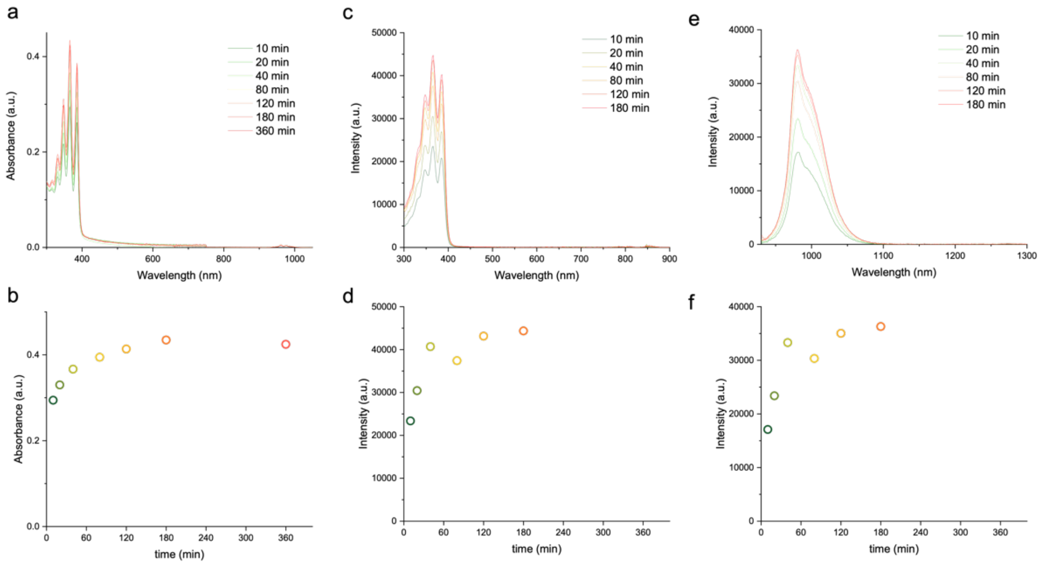

Supplementary Fig. 8 | Effect of ligand exchange time. a, Absorption spectra of YbNP@9-ACA nanohybrids collected at different ligand exchange times and **b,** corresponding peak absorption intensity change versus ligand exchange time. **c,** PLE spectra of YbNP@9-ACA nanohybrids collected at different ligand exchange times and **d,** corresponding the corresponding peak intensities at 364 nm. **e,** PL spectra of YbNP@9-ACA nanohybrids measured at different ligand exchange times and **f,** corresponding peak emission intensity change with ligand exchange time.

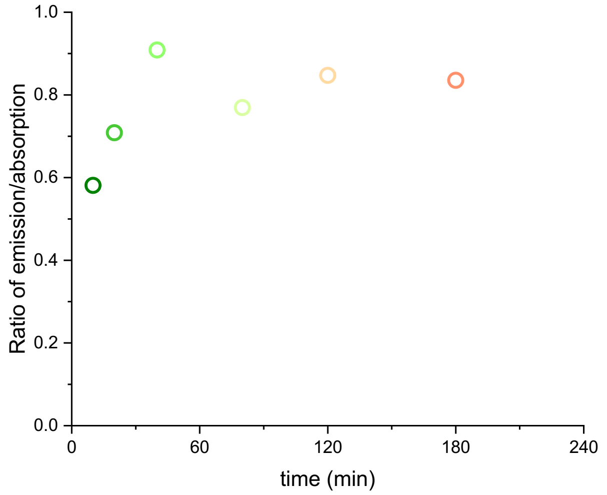

Supplementary Fig. 9 | The ratio of peak emission intensity to the absorption of YbNP@9-ACA nanohybrids versus ligand exchange time.

Supplementary Table 4 Quantum efficiencies of LnNPs and LnNP@9-ACA nanohybrids.

Sample Name	NdNPs	NdNP@9-ACA
QE	0.02%	0.39%
Sample Name	YbNPs	YbNP@9-ACA
QE	0.02%	0.14%
Sample Name	ErNPs	ErNP@9-ACA
QE	0.01%	0.03%

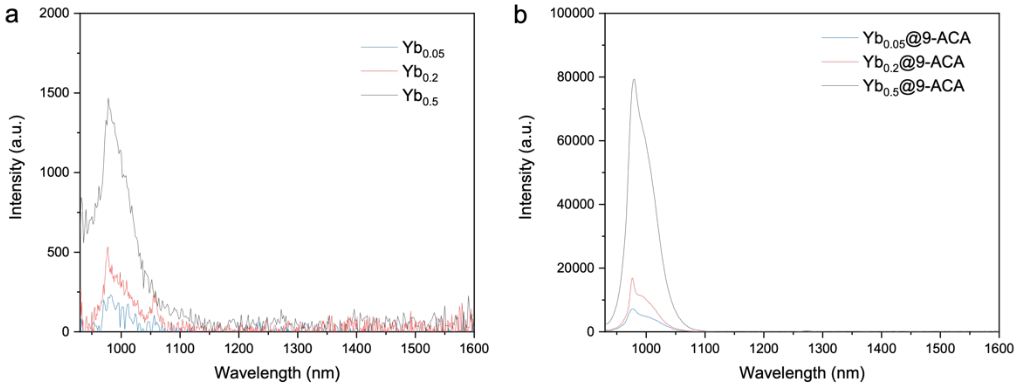

Supplementary Fig. 10 | Effect of Ln^{3+} doping concentration on NIR emission. NIR PL spectra of **a,** YbNPs with different doping concentrations and **b,** corresponding YbNP@9-ACA nanohybrids (concentration of 20 mg ml^{-1}) under 350 nm excitation.

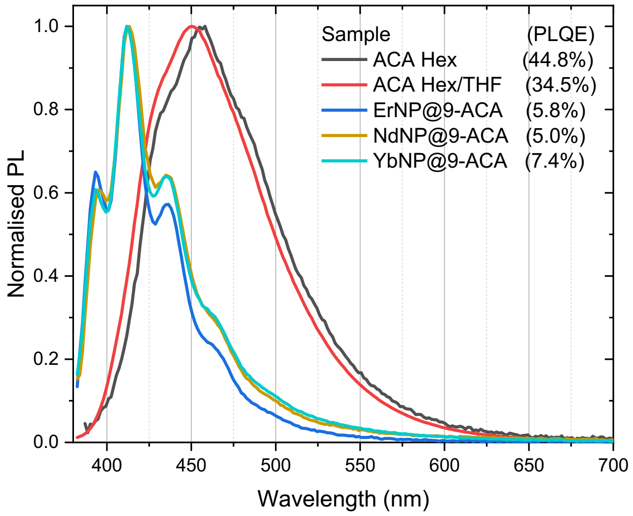

Supplementary Fig. 11 | PLQEs of 9-ACA in different solvents and different LnNP@9-ACA nanohybrids in visible range.

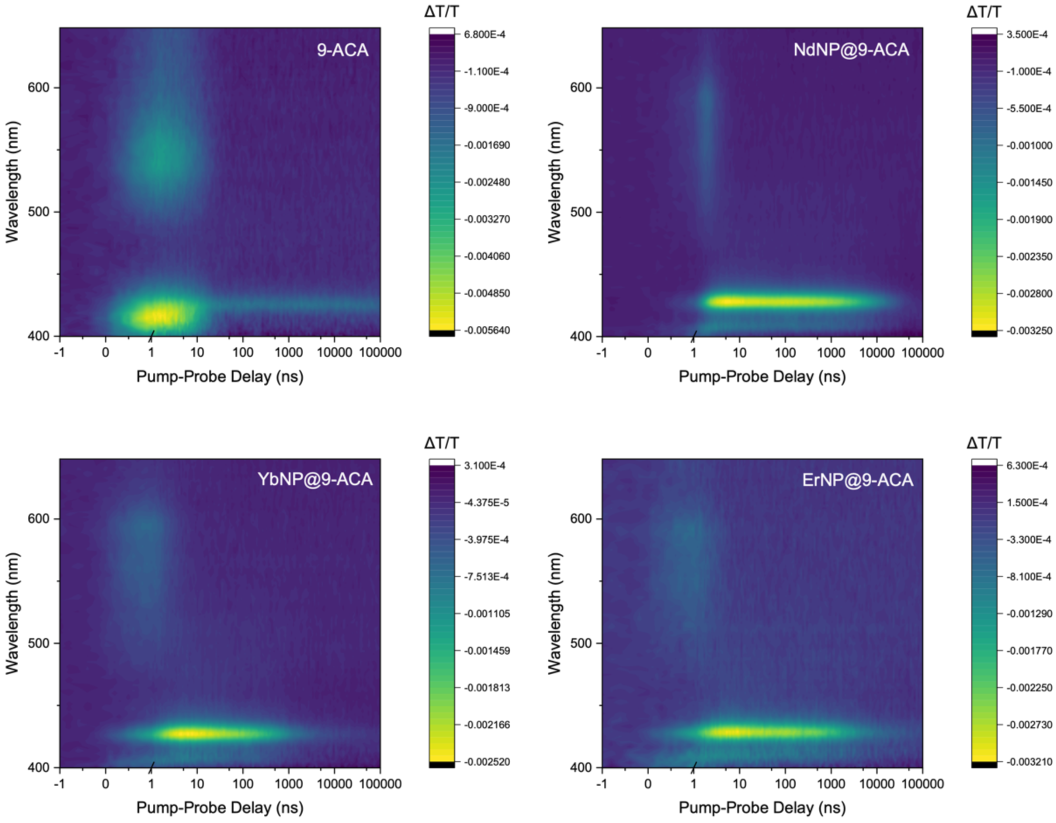

Supplementary Fig.12 | TA mapping of pristine 9-ACA and LnNP@9-ACA nanohybrids.

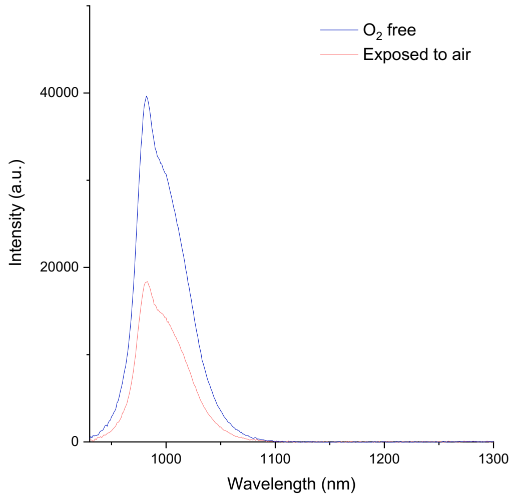

Supplementary Fig.13 │ PL intensity change of nanohybrids exposed to air. NIR PL spectra of YbNP@9-ACA nanohybrids (concentration of 20 mg ml^{-1}) under 350 nm excitation show significant quenching in emission intensity of Yb^{3+} after exposure to air.

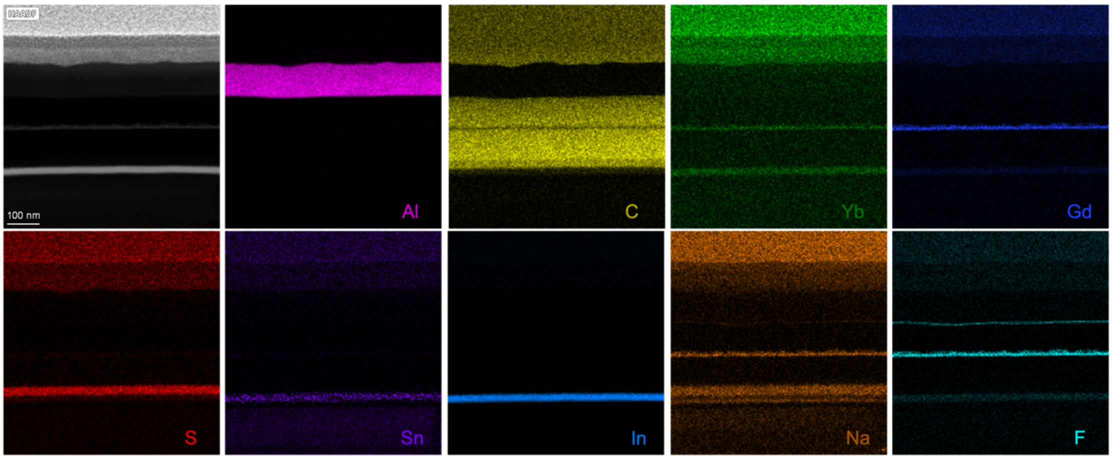

Supplementary Fig.14 | HAADF STEM element mapping of the cross-section of YbLEDs.

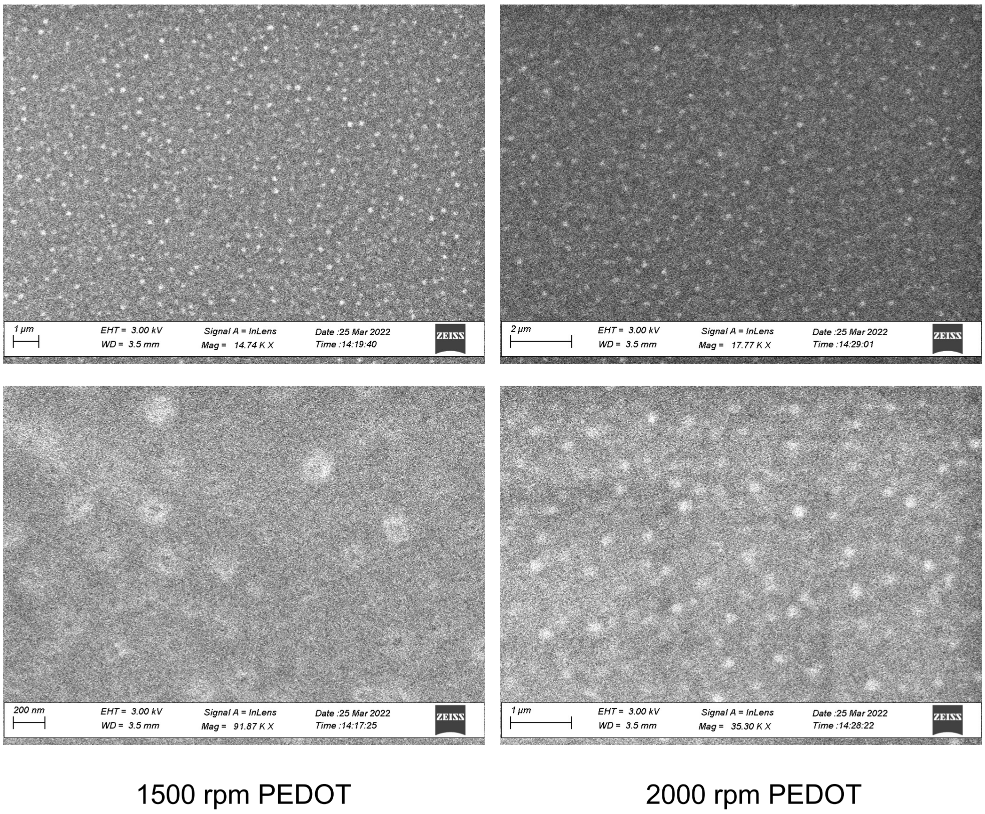

Supplementary Fig.15 | SEM images of NdNP@9-ACA on PEDOT/ITO substrates.

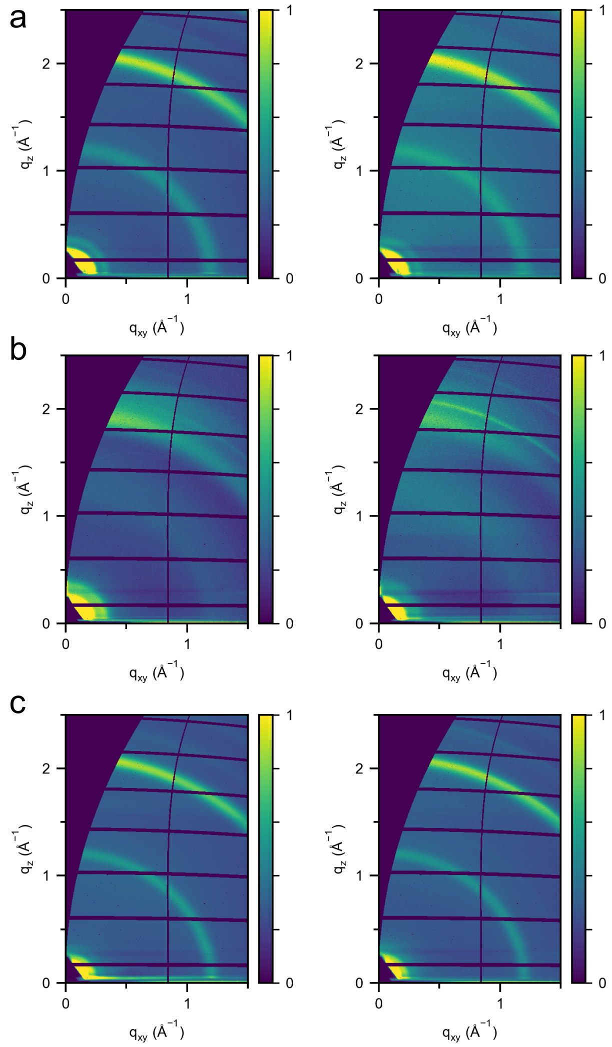

Supplementary Fig. 16 | GIWAXS measurements of LnNPs and LnNP@9-ACA nanohybrids. a, NdNP@OA and NdNP@9-ACA; **b,** YbNP@OA and YbNP@9-ACA; **c,** ErNP@OA and ErNP@9-ACA on poly-TPD/PEDOT:PSS/ITO substrates.

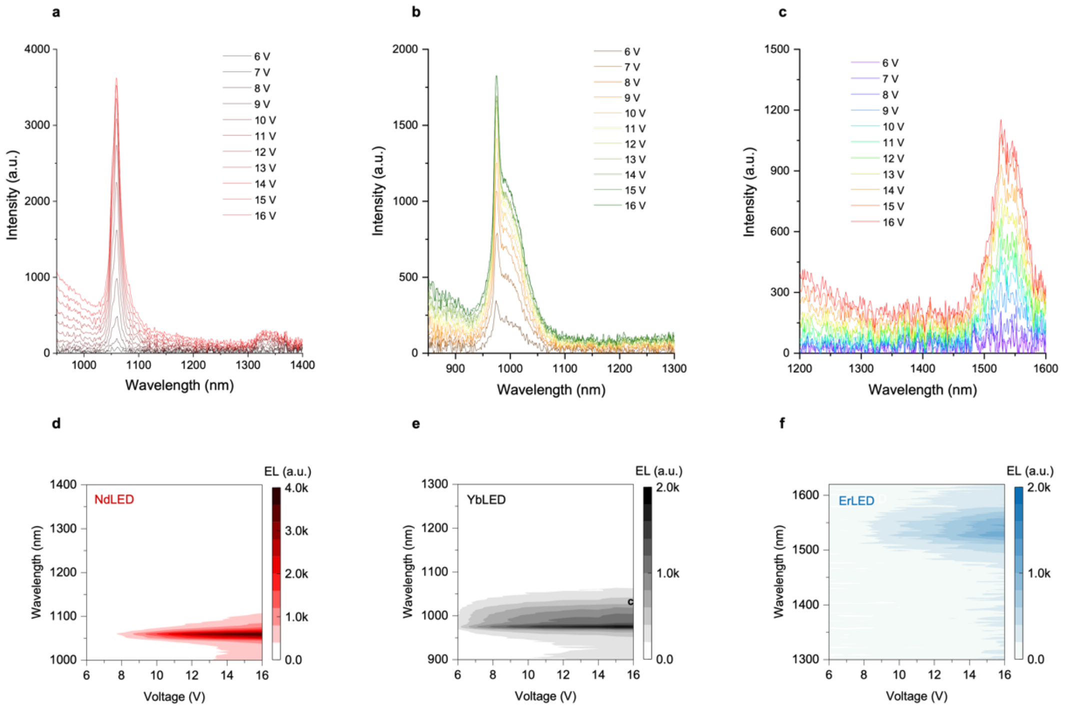

Supplementary Fig. 17 │ NIR EL spectra versus voltage and corresponding EL mapping of LnLEDs. a, NdNP-LED; **b,** YbNP-LED; and **c,** ErNP-LED. EL mapping of **d,** NdNP-LED; **e,** YbNP-LED; and **f,** ErNP-LED at different voltages.

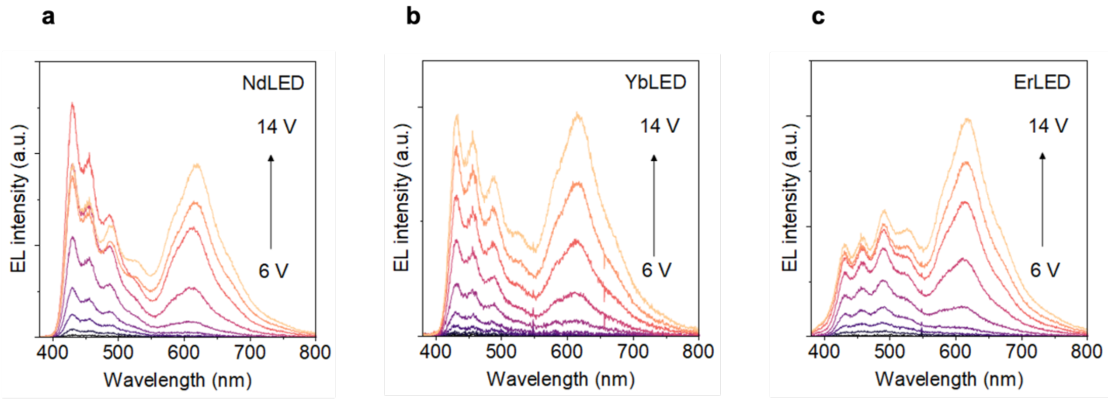

Supplementary Fig. 18 | Visible EL spectra versus voltage for LnLEDs. a, NdLED, **b,** YbLED, and **c,** ErLED. The EL peak at ~610 nm resembles that of the exciplex formed at the interface of poly-TPD and organic ETL (Yin, Y. et al., Journal of Materials Science: Materials in Electronics 28, 19148-19154, (2017)). This feature indicates the electron-hole recombination at the direct contact of HTL and ETL, in voids of our monolayer LnNP@9-ACA hybrids, without forming excitons on the hybrids. The EL features at ~430 nm corresponds to the spectra of poly-TPD, indicating that excitons are formed inside the HTL due to electron overflow from the emitting layer.

Supplementary Note 2 LED Characterization.

A calibrated Germanium power meter with known spectral responsivity (A W^{-1} nm^{-1}), $R(\lambda)$, was used to characterize the photon flux and radiance of the EL in the NIR region (see Supplementary Fig. 23 for the detector properties). The visible emission was filtered out by using a long-pass filter (950 nm for Nd and Yb-LEDs, 1300 nm for Er-LEDs) attached to the power meter. The detector head (10×10 mm^2) was placed in the normal direction of the device plane (2×2 mm^2). The distance between the detector and the LED (15 mm) was more than five times larger than the device area to fulfill the spot light-source assumption for the goniometric measurement[1].

The effective NIR responsivity of the power meter in a measurement (R_{EL}, in A W^{-1}) was calculated according to $R(\lambda)$ and the NIR-EL spectrum (W nm^{-1}) of the testing LED, $EL(\lambda)$:

$$R_{EL} = \frac{\int R(\lambda)\, EL(\lambda)\mathrm{d}\lambda}{\int EL(\lambda)\mathrm{d}\lambda}$$

The total NIR radiant flux (*P*) of the LED was calculated from the measured photo current (*I*) and the geometric factor (*G*) of the set-up:

$$P = \frac{I \cdot G}{R_{EL}}$$

The geometric factor, G, represents the fraction of the collected light (within a solid angle of Ω_M) with respect to the total light emitting into the forward hemisphere (within a solid angle of $\Omega_{2\pi}$) of the LED, and was calculated by:

$$G = \frac{\int_{\Omega_{2\pi}} I(\theta) d\Omega}{\int_{\Omega_M} I(\theta) R(\theta) d\Omega} = \frac{\int_0^{\pi/2} I(\theta) sin\,(\theta) d\theta}{\int_0^{\theta_M} I(\theta) R(\theta) sin\,(\theta) d\theta}$$

where θ is the emitting angle respect to the normal direction of the device plane. The largest collecting angle, θ_M, was determined by the detection distance. $I(\theta)$ and $R(\theta)$ are the angular dependent EL intensity of the LED and the angular dependent responsivity of the power meter, respectively. The angular dependency of EL from the LnLED was assumed to be a Lambertian profile. Schematic diagram on the light collection geometrics can be found in Supplementary Fig. 19.

The NIR radiance of the LED (L, in W sr^{-1} m^{-2}) was calculated according to the Lambertian assumption:

$$L = \frac{P}{\pi \cdot A_{LED}}$$

where A_{LED} is the device area.

The total NIR photon flux (N_{ph}) of the LED was calculated by considering the total radiant flux and the average photon energy of the NIR EL spectrum:

$$N_{ph} = P / \frac{\int EL(\lambda)\mathrm{d}\lambda}{\int EL(\lambda) / \frac{hc}{\lambda}\mathrm{d}\lambda}$$

where h and c are Planck constant and speed of light, respectively.

The EQE at a driving current (I) was calculated by:

$$EQE = \frac{N_{ph}}{I/e}$$

where e is elementary charge.

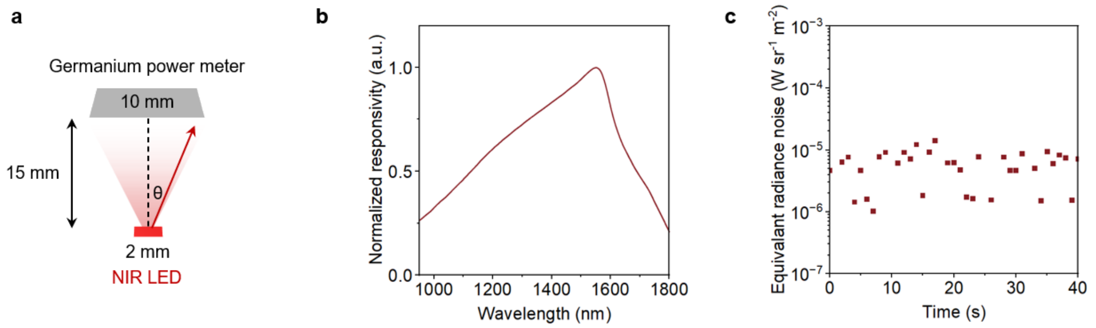

Supplementary Fig. 19 | Efficiency measurement of NIR LEDs. a, Schematic diagram of the light-detection configuration. A geometric calibration was performed to obtain the total radiant flux of the LED from the collected radiant flux. **b,** Normalized spectral responsivity of the calibrated Germanium power meter in NIR region. **c,** Equivalent radiance from the background noise of the LED measurement system, which was measured on a ErLED with the active area covered by a non-transparent tape. The result indicates the decent signal-to-noise ratio of the system that allows the effective measurement on radiance of ~10 μW sr^{-1} m^{-2}.

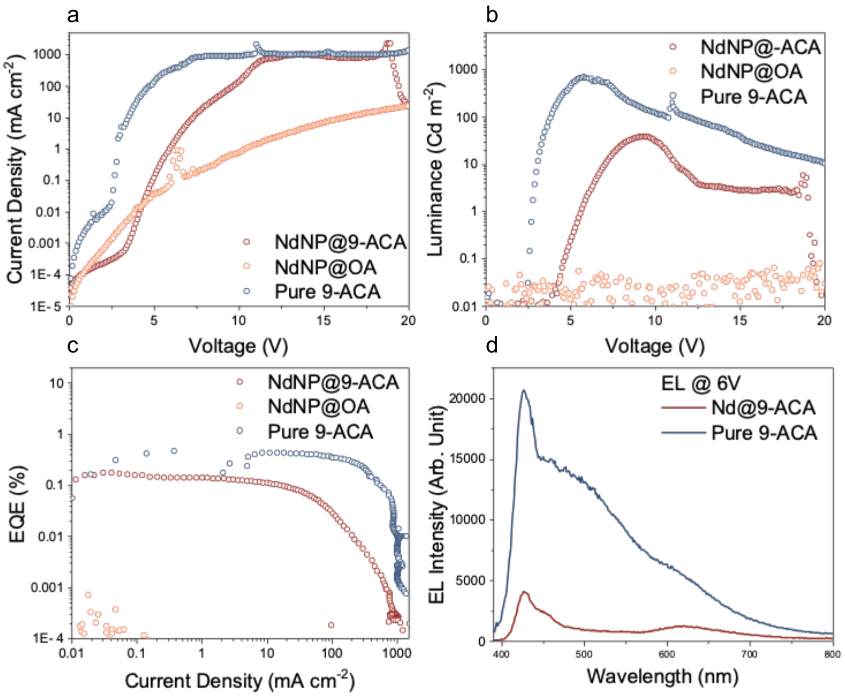

Supplementary Fig.20 | Characterization of NdNP@OA, NdNP@9-ACA and pure 9-ACA LEDs. **a,** Voltage- current density of NdNP@OA, NdNP@9-ACA and pure 9-ACA LEDs. **b,** Voltage- luminescence of NdNP@OA, NdNP@9-ACA and pure 9-ACA LEDs. **c,** Visible range current density- EQE of of NdNP@OA, NdNP@9-ACA and pure 9-ACA LEDs. **d,** EL of dNP@9-ACA and pure 9-ACA LEDs at 6V.
